# What is the influence of tibial component posterior slope on clinical and radiographic outcomes following cemented medial unicompartmental fixed-bearing knee arthroplasty? A retrospective study with a minimum follow-up of five years

**DOI:** 10.1007/s00264-025-06579-0

**Published:** 2025-06-25

**Authors:** Maksym Polt, Titus Thut, David Alexander Graf, Naeder Helmy, Octavian Andronic

**Affiliations:** 1 Balgrist University Hospital, Zürich, Switzerland; 2 Bürgerspital Solothurn, Solothurn, Switzerland

**Keywords:** Tibial slope, Posterior tibial slope, Tibial component positioning, Unicompartmental knee arthroplasty, Unicompartmental knee replacement, Aseptic loosening, Tibial aseptic loosening, Periprosthetic radiolucency, Tibial periprosthetic radiolucency

## Abstract

**Purpose:**

To evaluate how changing the native posterior tibial slope (PTS) through implantation of a cemented medial unicompartmental knee arthroplasty (UKA) impacts clinical and radiographic outcomes, specifically whether it correlates with the occurrence of tibial periprosthetic radiolucency or tibial aseptic loosening (AL).

**Methods:**

This retrospective study analyzed 63 patients with cemented medial UKAs with a minimum follow-up of five years. Patient-reported outcomes (PROMs) included the Oxford Knee Score (OKS). Radiographic parameters assessed were: PTS, mechanical axis, prosthetic joint space height, tibial component obliquity, intraprosthetic divergence, and tibial periprosthetic radiolucency. Partial Pearson correlation and multiple linear regression analyses were used to evaluate the relationship between tibial periprosthetic radiolucency and demographic or radiographic parameters.

**Results:**

Of 63 patients (mean age 68.9 ± 7.9 years, follow-up 62.5 ± 8.8 months), 5 knees (7.9%) demonstrated tibial periprosthetic radiolucency ≥ 2 mm. The mean postoperative PTS change was 3.8 ± 2.6°, mechanical axis change: 2.5 ± 1.8°, prosthetic joint space height: 9.2 ± 3.1 mm, tibial component obliquity: 2.5° ± 3°, and intraprosthetic divergence angle: 5° ± 4°. OKS averaged 43.9 (range 22–48), with a mean knee flexion of 123.4 ± 6.8°. Statistical analysis showed no significant associations between tibial periprosthetic radiolucency and demographics, radiographic parameters, or PROMs. Changes in PTS did not correlate with a range of motion (ROM), PROMs, or radiolucency.

**Conclusion:**

In our cohort, the deviation from native PTS following implantation of the cemented tibial component did not show a significant correlation with tibial periprosthetic radiolucency, PROMs, or ROM at mid-term follow-up.

## Introduction

In isolated medial or lateral osteoarthritis (OA) of the knee, unicompartmental knee arthroplasty (UKA) is an excellent alternative to total knee arthroplasty (TKA) with promising long-term results [1, 2]. In contrast to TKA, UKA is associated with fewer complications and mortality [3], as well as an increased range of motion due to the preservation of kinematically important structures such as the cruciate ligaments [4]. Despite a ten year survival rate exceeding 95% [5], UKA has higher revision rates than TKA [6]. Aseptic loosening (AL) is a leading cause of failure for cemented implants, often identified radiographically by progressive periprosthetic radiolucent lines (RLL) beneath the tibial component [6–9]. Among factors discussed to improve implant longevity, tibial component positioning, particularly posterior tibial slope (PTS), has been linked to AL in cemented UKAs [10, 11]. Prior studies have also associated implant survival with mechanical axis alignment and joint space preservation [11–15].

Pandit et al. reported fewer RLLs in uncemented versus cemented UKAs after one year, suggesting better bone ingrowth with cementless implants [16]. This supports the notion that tibial component positioning may be especially critical in cemented UKA, though the role of PTS remains debated [10, 11, 17].

The primary objective of this study is to evaluate the impact of change in PTS through implantation of a cemented medial UKA on the incidence of tibial periprosthetic radiolucency, the incidence of tibial AL, and the clinical outcomes following cemented medial UKA. The secondary objective is to assess the influence of additional factors, including mechanical axis, prosthetic joint space height, tibial component obliquity, and intraprosthetic divergence, on the occurrence of tibial periprosthetic radiolucency, incidence of tibial AL, and on the clinical outcomes following cemented medial UKA. We hypothesized that deviation in the PTS would exhibit the strongest correlation with increased tibial periprosthetic radiolucency, the incidence of tibial AL, and poorer clinical outcomes following cemented medial UKA.

## Materials and methods

This retrospective study was approved by the Ethics Commission board of Northwest and Central Switzerland (ID 2022 − 01320) and was conducted entirely at the author’s institution.

### Study cohort

Out of the institution’s database, which included every unicompartmental or total knee replacement executed between September 2011 and January 2019, all medial UKAs were identified. Patients with a minimum follow-up of five years and cemented components only were reviewed retrospectively. They had undergone primary medial UKA for symptomatic, medial unicompartmental knee OA stage III-IV according to the Kellgren–Lawrence classification (primary or secondary) [18] with various degrees of patellofemoral osteoarthritis (PFOA) (Kellgren-Lawrence 1–4) and sparing of the lateral tibiofemoral compartment. Preoperative radiological assessment was carried out by analyzing weight-bearing anteroposterior, lateral, and Rosenberg views of the knee and an axial view of the patella. The mechanical axis was evaluated preoperatively using a weight-bearing full-length anteroposterior radiograph.

Qualifying prerequisites consisted of frontal deformity < 15°; flexion contracture < 15°; functional integrity of the anterior cruciate ligament (ACL) and peripheral ligaments of the knee, as well as the absence of an inflammatory arthropathy [19]. Indication for UKA implantation for anteromedial OA required medial bone-on-bone arthritis, a functionally normal ACL, functionally normal collateral ligaments, and intact full-thickness lateral tibiofemoral cartilage [1].

### Surgical technique

All UKAs were performed by two staff surgeons using a minimally invasive medial parapatellar approach with patellar subluxation. Patient-specific, 3D-printed cutting guides based on preoperative CT scans were used for tibial and femoral preparation. Cemented, fixed-bearing implants by MyKnee Solutions of Medacta International^®^ (Strada Regina, 6874 Castel San Pietro, Switzerland) were used. In smaller tibias (size ≤ 2), full-polyethylene components may have been selected [20]. Clinical and radiological evaluations were performed at three months, one year, and five years postoperatively.

### Clinical outcome measures

At follow-ups, Oxford Knee Score (OKS) as a representative of the patient-reported outcomes (PROMs) [21] and range of motion (ROM) as an objective value for knee function were assessed in a standardized way. Only values from the final consultation were analyzed. Cases with missing ROM data were excluded. Revision surgeries for tibial AL were documented, defined as knee pain with pathological tibial radiolucency (≥ 2 mm, poorly defined, progressive) on anteroposterior radiographs, with infection ruled out by aspiration [7].

### Radiographic assessment

Pre- and postoperative weight-bearing radiographs were used to evaluate medial and lateral tibiofemoral degeneration (Kellgren-Lawrence classification), mechanical axis, PTS, prosthetic joint space height, intraprosthetic divergence, tibial component obliquity, and tibial periprosthetic radiolucency. All measurements were taken from the last follow-up (≥ 5 years post-op) and analyzed in a blinded fashion using the picture archiving and communication system (PACS) tools.

### Mechanical axis of the lower limb

Mechanical axis measurements were performed on full-length weight-bearing anteroposterior radiographs [22]. A hip-knee-ankle angle (HKA), measuring the angulation between a line from the centre of rotation of the femoral head to the femoral intercondylar notch and a line from the tibial intercondylar eminence to the tibial mid-plafond, was assessed. The difference between the pre- and postoperative measurements was calculated and defined as a *delta mechanical axis*. Valgus alignment of the knee, where the patellar center lay medial to the Mikulicz line, was defined as a negative value. Varus alignment was assigned a positive value.

### PTS

**Fig. 1:**
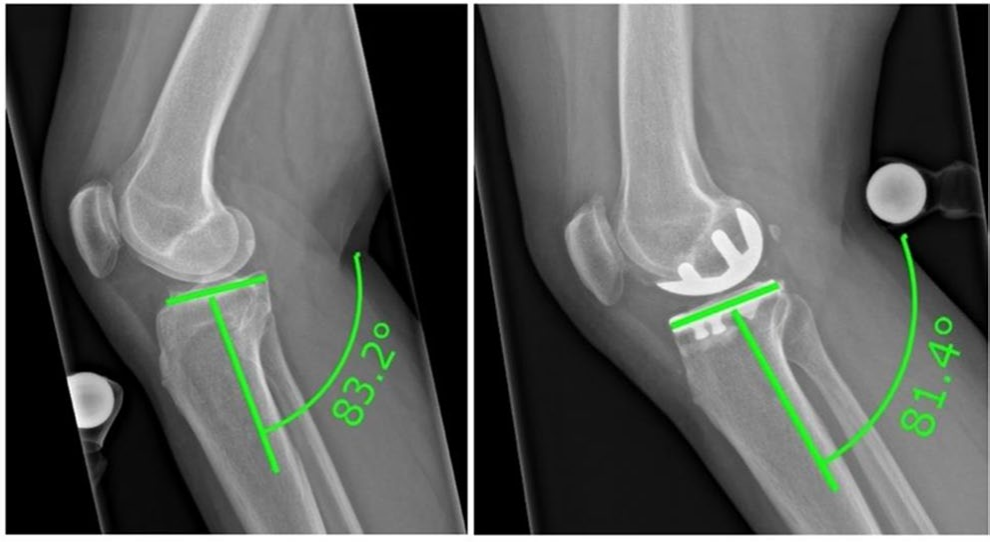
Measurement of the pre- and postoperative PTS

PTS was measured as an angle between a perpendicular line to the tibial diaphysis axis and the tangent of the most anterosuperior and posterosuperior points of the medial tibial plateau [23] (Fig. 1). The difference between the pre- and postoperative measurements was calculated and was defined as a *delta tibial slope*.

### Prosthetic joint space height

**Fig. 2:**
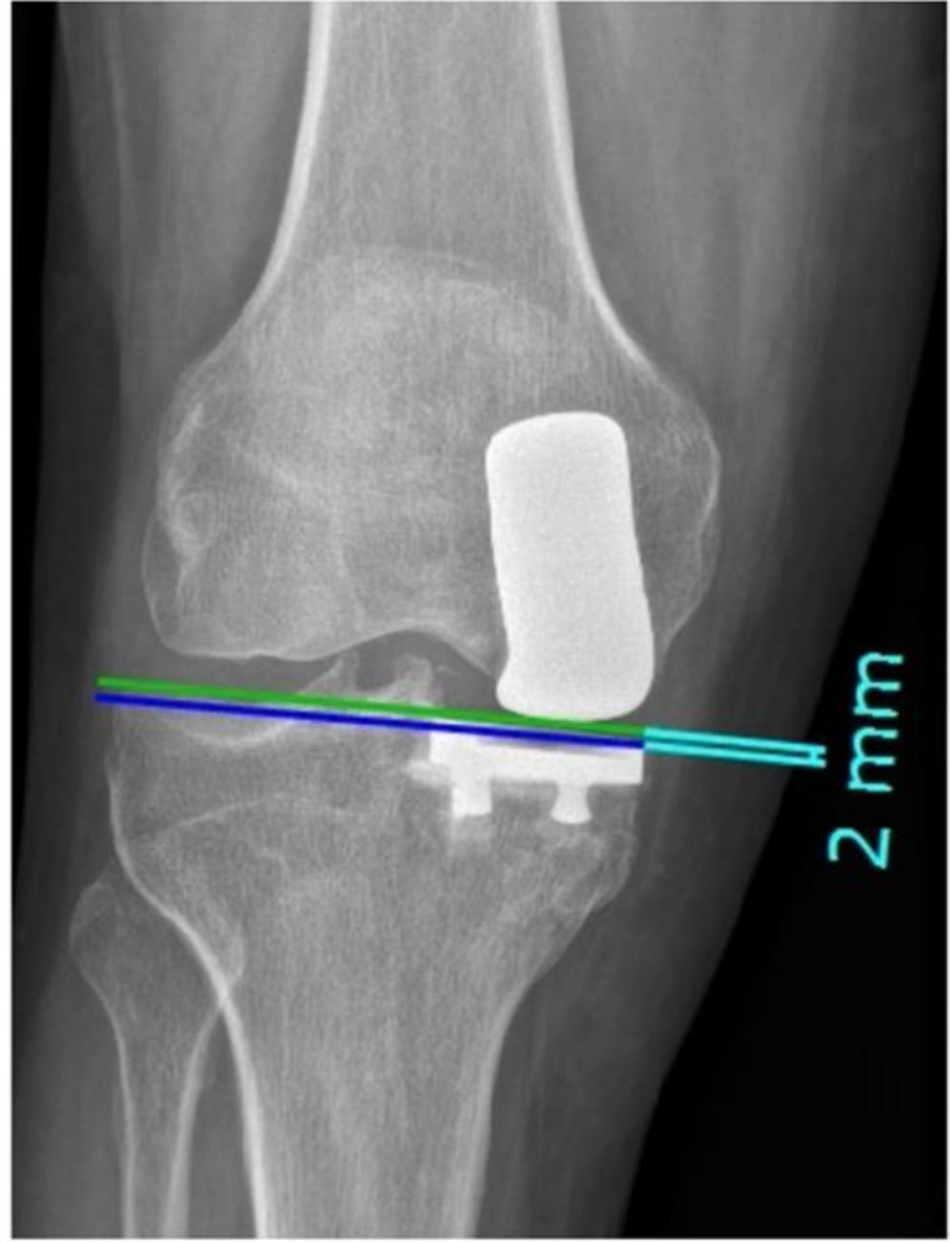
Measurement of the level of the prosthetic joint space

This factor quantifies the height difference between the tangent line to the articular surface of the tibial implant and the lateral femorotibial joint space [11] (Fig. 2). This way, the level of the prosthetic joint space can be assessed [11].

### Tibial component obliquity

**Fig. 3:**
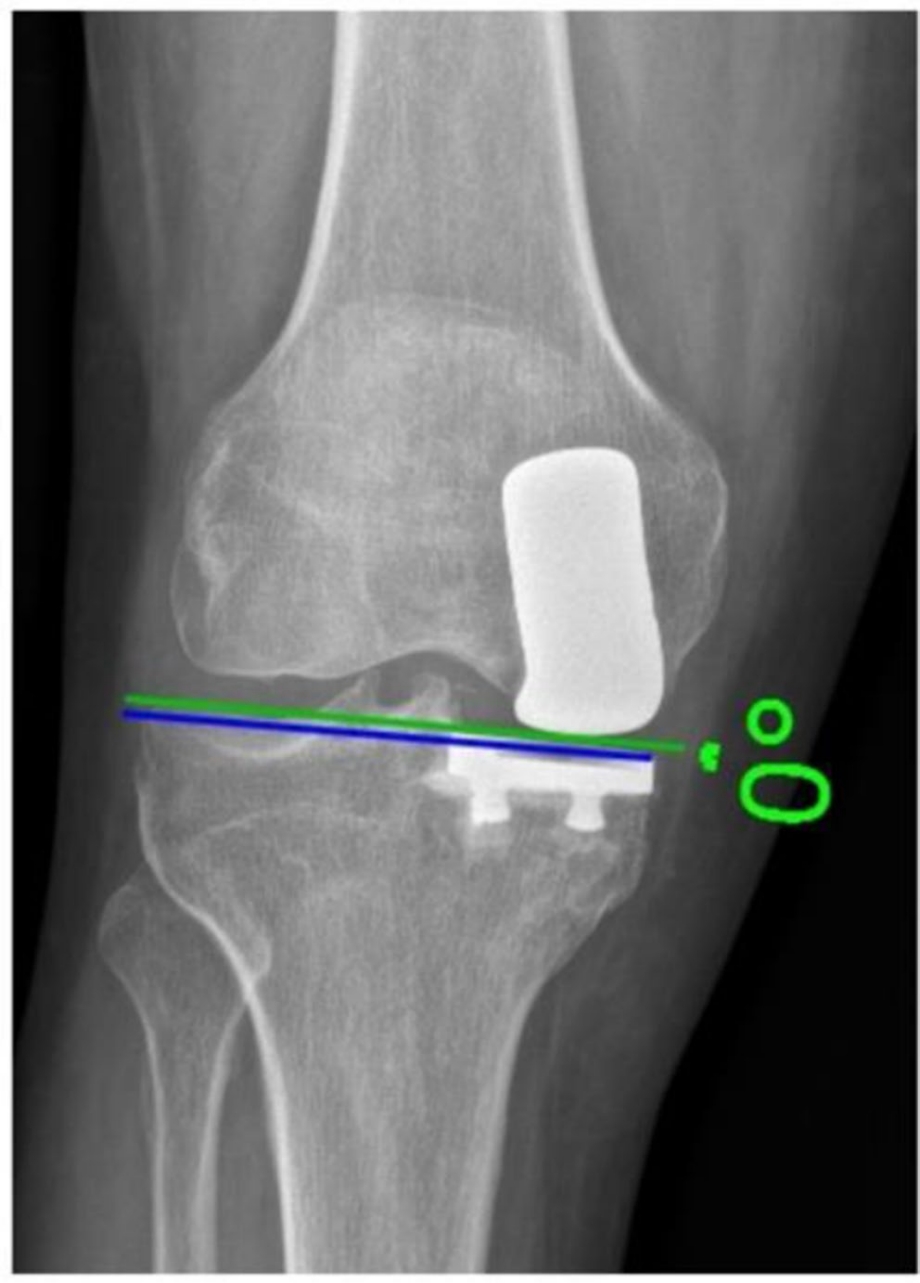
Measurement of the tibial component obliquity showing an alignment of 0° between the tibial component and lateral femorotibial joint space

It was assessed by measuring the angle formed between the tangent to the tibial component and the line extending along the lateral femorotibial joint space, with varus angles assigned positive values and valgus angles negative values [11] (Fig. 3).

### Intraprosthetic divergence

**Fig. 4:**
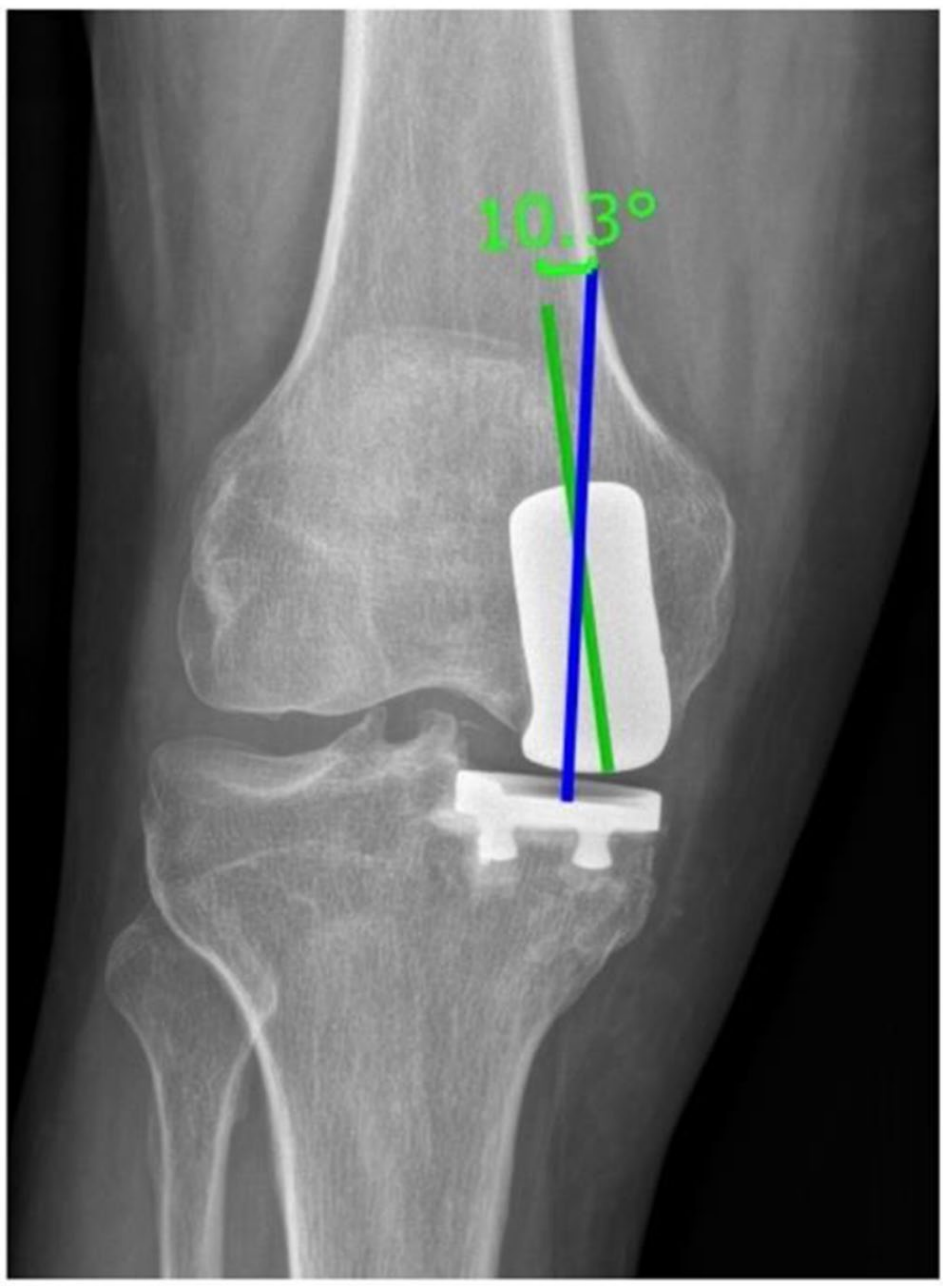
Measurement of the intraprosthetic divergence

To assess the intraprosthetic divergence from 90° in the coronal plane, the angle between the longitudinal axis of the femoral condyle and the line perpendicular to the tangent of the tibial implant was measured [11] (Fig. 4).

### Tibial periprosthetic radiolucency

**Fig. 5:**
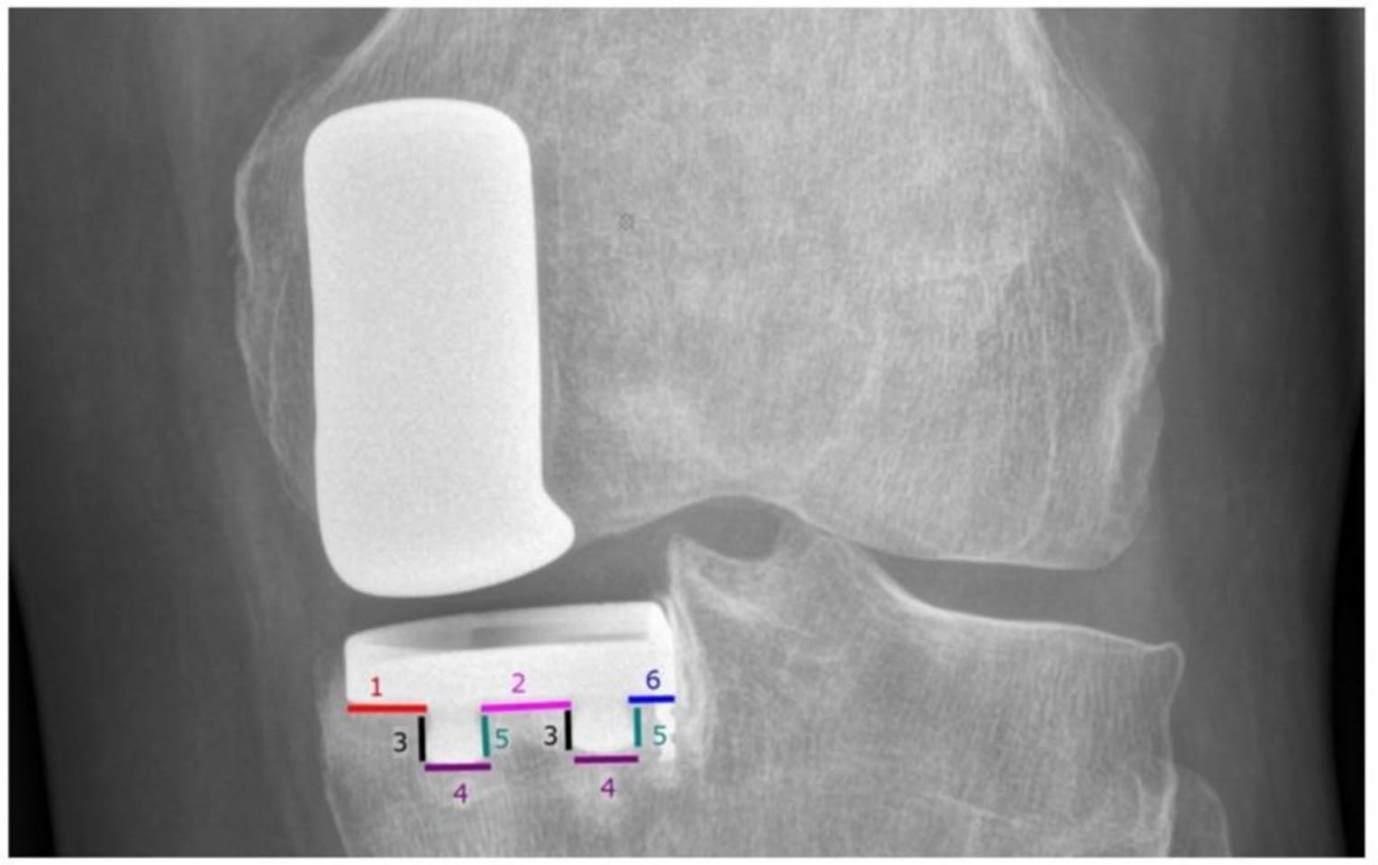
Six zones beneath the tibial component evaluated for radiolucency

In postoperative radiographs, the area beneath the tibial component was divided into six zones [7, 16] (Fig. 5). If radiolucency was detected, its area was measured perpendicular to the tibial component [7, 16], with areas of 2 mm or more considered relevant [7].

### Statistical analysis

Descriptive analysis was performed using means and standard deviations for continuous variables (independent t-test) and frequencies or percentages for discrete or dichotomous variables (Chi-squared or Fisher’s exact test). Partial Pearson correlation analysis was performed to search for correlations between the occurrence of tibial periprosthetic radiolucency with PROMs at the last follow-up (OKS).

For the analysis of independent radiographic parameters that might have influenced the occurrence of tibial periprosthetic radiolucency, irrespective of the region, a multiple linear regression analysis was used. First, the relevant demographic factors were included: body mass index (BMI), age, gender, and smoking status. The separate regression analysis included the relevant radiographic factors: delta tibial slope, delta mechanical axis, intraprosthetic divergence angle, prosthetic joint space height, and tibial component obliquity. An apriori power analysis (fixed model) for a medium effect size (f2) = 0.15 and a desired statistical power of 1 − β > 0.8 (total number of predictors: 5, 4 for the demographic analysis and 5 for the radiographic analysis) was performed to secure a sufficient sample size for the multiple regression analysis regarding demographic and radiographic predictors for tibial periprosthetic radiolucency. The minimum sample size of *n* = 55 was, therefore, achieved with the current study population (*n* = 63).

## Results

Records of 6 patients showed missing ROM, thus, these patients were excluded from further analysis. Out of the remaining 63 patients, 26 were female and 37 were males, with a mean age of 68.9 ± 7.9 years. The mean follow-up was 62.5 ± 8.8 months (range 60–108 months). During this follow-up period, only one revision had to be performed due to persistent anterior pain. The mean BMI was 28.9 ± 4.0.

Tibial periprosthetic radiolucency of 2 mm or more at the last follow-up was detected six times and in five knees (7.9%). The distribution across different regions is presented in Table 1. In two cases, the radiolucency and its extension were unchanged compared to the follow-up one year after surgery. In one case, the radiolucency expanded from 1 to 4 mm. In another three cases, a local radiolucency was observed for the first time at the five year follow-up. The mean postoperative delta tibial slope was 3,8 ± 2.6°. The postoperative slope change in cases with detected tibial periprosthetic radiolucency is presented in Table 1. The calculated mean values of the evaluated parameters are presented in Table 2. The OKS averaged 43.9 at the last follow-up (range 22–48). While the mean knee flexion in the investigated population was 123.4 ± 6.8°, two patients had extension deficits over 5°.

On the multiple regression analysis (R^2^ = 0.128 for demographics), male gender positively correlated with radiolucency occurrence with almost-significant statistical value (*p* = 0.053). Age (*p* = 0.519), smoking status (*p* = 0.764), and BMI (*p* = 0.147) were not independently associated with the occurrence of tibial periprosthetic radiolucency. The regression analysis that included the radiographic parameters (R^2^ = 0.107), could not reveal any variable that was associated with the occurrence of radiolucency at the last follow-up: delta tibial slope (*p* = 0.465), prosthetic joint space height (*p* = 0.236), intraprosthetic divergence angle (*p* = 0.063), delta mechanical axis (*p* = 0.621) and tibial component obliquity (*p* = 0.709).

There was no correlation between the occurrence of tibial periprosthetic radiolucency and the PROMs (OKS) at the last follow-up (*p* = 0.969, Pearson analysis). Delta tibial slope did not influence knee flexion (*p* = 0.562, Pearson analysis) or knee extension (*p* = 0.720, Pearson analysis).

**Table 1:**
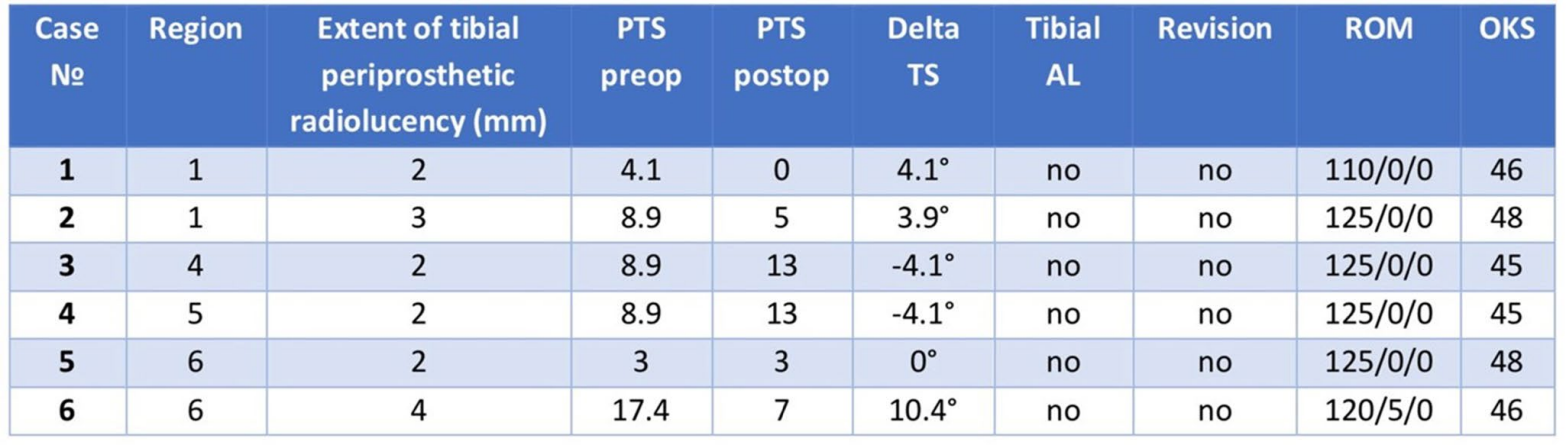
Six cases of tibial periprosthetic radiolucency of 2 mm or more with a demonstration of the exact region, the extent of radiolucency, preoperative and postoperative posterior tibial slope (PTS), and delta tibial slope (TS). Positive values stand for PTS decrease and negative for increase. The presence of tibial aseptic loosening (AL), the potential need for revision, range of motion (ROM), and Oxford knee score (OKS) are also demonstrated

**Table 2:**
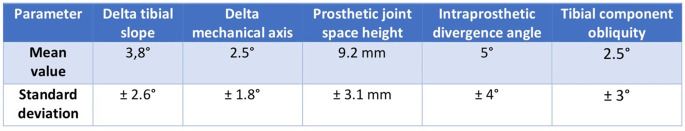
Parameters evaluated during radiographic assessment with their mean values and standard deviation. The tibial component obliquity was given positive values for varus angles and negative for valgus angles

## Discussion

Our study found no association between tibial periprosthetic radiolucency and postoperative deviation from native PTS. Moreover, we did not find any cases of tibial AL in the investigated population. The only revision that had to be performed was due to persistent anterior knee pain. Despite six cases of tibial periprosthetic radiolucency over 2 mm, no correlation could be established between the radiolucency and the PROMs (OKS). Interestingly, the deviation of the tibial component slope did not influence knee flexion or extension. These results contrast with prior findings that suggested high postoperative PTS or its significant alteration in comparison to the preoperative value could significantly influence outcomes in UKA. In the study by Chatellard et al., excessive PTS (> 5°) or a change in PTS beyond 2° from the preoperative value was associated with decreased prosthesis survival and mechanical failure [11]. In contrast, the mean delta tibial slope in our population was 3,8 ± 2.6°. Similarly, Hernigou and Deschamps demonstrated that a postoperative PTS exceeding 7° was associated with implant loosening, especially in knees with absent or compromised anterior cruciate ligaments (ACL) [10]. Unlike in our study, no radiologic evaluation of the tibial periprosthetic radiolucency was performed in the mentioned papers. Our study is the first to investigate the association between tibial periprosthetic radiolucency and delta tibial slope.

Prosthetic joint space height, intraprosthetic divergence angle, delta mechanical axis, or tibial component obliquity showed no correlation with tibial periprosthetic radiolucency or PROMs. Our finding considering the prosthetic joint space height contrasts with findings from Kamenaga et al., who reported a significant negative correlation between tibial component height (TCH) and the Oxford Knee Score (OKS) at two years postoperatively, suggesting that lower TCH relative to the lateral compartment adversely affects clinical outcomes [24]. Additionally, Chatellard et al. identified joint space elevation greater than 2 mm as a risk factor for reduced prosthesis survival, emphasizing that maintaining prosthetic joint space height within 3 mm of the lateral compartment is critical for longevity [11]. Also, the findings considering the intraprosthetic divergence angle contrast with the findings of Chatellard et al. [11], who identified that an intraprosthetic divergence angle exceeding 6° significantly increased the risk of mechanical failure in medial UKA. Our findings suggest that moderate divergence within a range of 5 ± 4° may not significantly compromise implant integrity. No significant association was observed between the delta mechanical axis and the occurrence of tibial periprosthetic radiolucency aligns with the findings of Zuiderbaan et al. [13] who found that patients with a postoperative mechanical axis alignment of 1°–4° varus experienced superior clinical outcomes compared to those with more extreme alignments. Similarly, Chatellard et al. [11] identified residual mechanical varus of 5° or more as a significant risk factor for mechanical failure in medial unicompartmental knee arthroplasty (UKA). These results suggest that maintaining limb alignment within a neutral range is critical for implant longevity. While Chatellard et al. stated that a change in tibial component obliquity greater than 3° in varus direction significantly reduced prosthesis survival [11], we could not find any association between tibial periprosthetic radiolucency and the mean tibial component obliquity of 2.5 ± 3°.

In our study, male gender showed a nearly significant positive correlation with the occurrence of tibial periprosthetic radiolucency (*p* = 0.053), while age (*p* = 0.519), smoking status (*p* = 0.764), and BMI (*p* = 0.147) did not independently correlate with tibial periprosthetic radiolucency, tibial AL, and therefore need for revision. These findings partially align with Aleto et al. [25], who reported that BMI and sex were not significantly linked to medial tibial collapse (MTC) in their cohort. In contrast, age was significantly associated with MTC, with older patients being more prone to this failure mode [25]. Interestingly, Collier et al. found that younger patient age significantly increased the risk of revision, while sex and weight were not associated with higher revision rates [15]. Their study highlighted that a ten year decrease in age doubled the odds of revision, emphasizing age as a critical factor for implant longevity [15].

While patient-specific 3D-printed cutting guides were used for every UKA in our cohort, no robotic system was employed. A 2024 meta-analysis of > 3000 cases found that robotic UKA reduced complication and revision rates compared with conventional instrumentation while maintaining comparable radiological accuracy [26]. Complementary prospective data from a 2024 MAKO-based series showed the planned PTS was reproduced within 0.6 ° ± 1.2°, keeping every implant inside a ± 1° target band and without increasing tibial radiolucency [27]. Collectively, these results suggest that although the moderate slope deviations observed in our cohort appeared clinically benign, robotic assistance may further minimize outliers and thereby lower the risk of implant failure.

In our opinion, tibial periprosthetic radiolucency represents a reliable parameter for the evaluation of the radiologic outcome, especially tibial AL in UKA. During the last few years, this parameter has been extensively investigated for the assessment of radiographic outcomes [28–30]. Moreover, Behrend et al. were able to demonstrate robust inter- and intra-observer reliability for assessment of the anteroposterior tibial view [30].

Our study had several limitations. Eight patients lacked postoperative OKS, and nine were missing pre- or postoperative mechanical axis measurements. These cases were excluded from the analyses. Preoperative OKS was not available, preventing assessment of OKS improvement. Tibial periprosthetic radiolucency was evaluated only in two dimensions on the anteroposterior view. In this context, it is important to acknowledge that plain radiographs may lack the sensitivity and reproducibility of three-dimensional CT, because even slight limb rotation during the radiological assessment can alter the apparent PTS by several degrees. Although our sample size and follow-up were smaller than in the study by Chatellard et al. [11], the same implant was examined, and all procedures were performed by just two experienced surgeons.

## Conclusion

In our cohort, the amount of deviation from native PTS following implantation of the cemented tibial component did not show a significant correlation with tibial periprosthetic radiolucency, PROMs, or ROM at mid-term follow-up. Further parameters like prosthetic joint space height, intraprosthetic divergence angle, delta mechanical axis, and tibial component obliquity did not show any correlation with tibial periprosthetic radiolucency or PROMs either.

## Data Availability

The datasets generated and analyzed during the current study are available from the corresponding author on reasonable request.
